# Nutritional and Metabolic Imbalance in Keratoconus

**DOI:** 10.3390/nu14040913

**Published:** 2022-02-21

**Authors:** Romina Mayra Lasagni Vitar, Filippo Bonelli, Paolo Rama, Giulio Ferrari

**Affiliations:** Cornea and Ocular Surface Disease Unit, Eye Repair Lab, IRCCS San Raffaele Scientific Institute, 20132 Milan, Italy; lasagnivitar.romina@hsr.it (R.M.L.V.); bonelli.filippo@hsr.it (F.B.); rama.paolo@hsr.it (P.R.)

**Keywords:** keratoconus, nutrients, metabolites, imbalance

## Abstract

Keratoconus (KC) is a progressive corneal degeneration characterized by structural changes consisting of progressive thinning and steepening of the cornea. These alterations result in biomechanical weakening and, clinically, in vision loss. While the etiology of KC has been the object of study for over a century, no single agent has been found. Recent reviews suggest that KC is a multifactorial disease that is associated with a wide variety of genetic and environmental factors. While KC is typically considered a disease of the cornea, associations with systemic conditions have been well described over the years. In particular, nutritional and metabolic imbalance, such as the redox status, hormones, metabolites, and micronutrients (vitamins and metal ions), can deeply influence KC initiation and progression. In this paper, we comprehensively review the different nutritional (vitamins and minerals) and metabolic (hormones and metabolites) factors that are altered in KC, discussing their possible implication in the pathophysiology of the disease.

## 1. Introduction

Keratoconus (KC) is the most common ectatic degeneration of the cornea. It is characterized by corneal thinning and steepening, which result in irregular astigmatism and vision loss. Initial presentation typically occurs during infancy/adolescence and usually progresses for 10 to 20 years, after which the disease is generally stable [1].The prevalence varies widely across the globe, from 0.2/100,000 in Russia to 4790/100,000 in Saudi Arabia [2], with an estimated global prevalence of 1.38/1000 [3]. Pediatric/young adult populations usually display a higher prevalence and a more aggressive form of disease, as opposed to later-onset KC [4,5,6]. Moreover, it seems that the genetic background predisposes some ethnicities to KC [7].

The pathogenesis of KC is an object of active debate. While no single etiologic factor has been identified so far, it is generally thought that KC is a multifactorial disease, which requires a genetic predisposition *and* an environmental factor to develop [8,9]. The role of genetic predisposition is supported by the observation that consanguinity is a risk factor for KC; a positive family history has been reported in 5–20% of KC cases [9,10]. Moreover, the prevalence of KC in first-degree relatives increases to 67% [11]. The exact nature of those genetic and environmental factors remains unknown with limited exceptions [12].

Notably, KC is generally considered an isolated ocular disorder. In fact, there is no clinical evidence of associated systemic diseases. There are, however, some exceptions; atopy [13], sleep apnea [14], mitral valve prolapse [15,16], hypothyroidism [17], and Down’s syndrome [18,19] have been detected in a percentage of KC patients varying from 21% to 70%. It is interesting to note that many of these diseases cause metabolic [20,21,22] and/or nutritional [22,23] imbalance. Therefore, it is reasonable to hypothesize that systemic nutritional and/or metabolic alterations may also play a role in KC pathogenesis. However, this is still a matter of debate, and a solid causal relationship has never been demonstrated.

Lastly, the fact that KC invariably affects both eyes, albeit to a different degree, points to a systemic etiology [24].

In this paper, we comprehensively review the different nutritional (vitamins and minerals) and metabolic (hormones and metabolites) factors that are altered in KC, discussing their possible implication in the pathophysiology of the disease.

## 2. Pathogenesis of Keratoconus

The structural alterations observed in KC corneas affect the epithelium, the epithelial basal membrane, the Bowman layer, and the stroma [25]. Morphological alterations of epithelial basal cells and degradation of extracellular matrix (ECM) occur [26], leading to thinning of the corneal stroma, keratocyte apoptosis, and nerve damage [27,28].

The initiation and progression of KC are deeply influenced by several nutritional and metabolic factors, such as inflammatory mediators, redox status, hormones, metabolites, and micronutrients (vitamins and metal ions) [29]. In fact, ECM remodeling is significantly influenced by these biochemical factors. For instance, metal ions such as copper and iron, and vitamins (e.g., vitamin C) are essential cofactors of enzymes involved in collagen synthesis and crosslinking [30].

In addition, chronic eye rubbing and allergies, well-known risk factors for KC [9], are associated with increased expression of metalloproteinases and proinflammatory mediators, which can favor collagen degradation and KC progression [31].

## 3. Nutritional and Metabolic Alterations in Keratoconus

An increasing body of evidence supports the role of different metabolic products and micronutrients in many ocular diseases, including KC [29,32,33]. In this section, we discuss the contribution of different nutrients, metabolites, and hormones in the development and progression of KC disease.

### 3.1. Vitamins

Vitamins are essential nutrients indispensable to many corneal functions. In recent years, the specific role of vitamin D (Vit D) in the maintenance of corneal integrity has been studied in detail [34]. Vit D maintains the corneal epithelial barrier function [35,36] and is needed for survival of endothelial cells [37]. In addition, anti-inflammatory and antimicrobial properties have been described [38]. Lastly, Vit D participates in the redox homeostasis as it has an antioxidant role [39].

Vit D is secreted into the tear fluid and the aqueous humor, from where it reaches the cornea [40,41]. In addition, the cornea possesses the molecular machinery to synthesize and activate native Vit D [36], after UV light activation, similarly to the epidermis.

The involvement of Vit D in KC pathogenesis was suggested several decades ago by Blackberg and Knapp [42]. They discovered that a Vit D-deficient diet was accompanied by ocular changes consistent with KC in preclinical animal models. Some years later, a preliminary study conducted in KC patients suggested that Vit D supplementation improves the disease [43], although interpretation of results is complicated by limited imaging techniques available at the time. The hypothesis that Vit D deficiency is common in KC patients was confirmed years later when it was shown that KC patients displayed lower serum levels of Vit D when compared to age-matched controls [44,45,46]. Interestingly, KC patients exhibit downregulation of proteins involved in Vit D transport (e.g., Vit D-binding protein) and activation [47]. Moreover, an association between Vit D deficiency and KC prevalence was suggested in people living in Middle East regions [46]. Although the serum level of Vit D was not associated with KC severity or progression rate, the probability of developing KC was significantly increased when the levels of Vit D were less than 10 ng/mL [44]. Taken together, these data suggest that Vit D plays a role in the pathogenesis of KC; however, the exact mechanism still needs further elucidation.

Vitamin C (Vit C) is a water-soluble antioxidant that is found at high concentrations in the tear film and the cornea. It is among the most potent antioxidant molecules in the human body and plays a key role in corneal wound healing [48] and ECM assembly (e.g., collagen synthesis) [49,50]. Moreover, Vit C strengthens the cornea by decreasing the distance between collagen fibrils and by enhancing crosslinking in vitro [51]. Interestingly, the levels of Vit C seem to be increased in KC corneas [52,53], suggesting an adaptive response to oxidative stress and impaired collagen synthesis. However, Vit C levels were not altered in the tears [54] or aqueous humor [52] of KC patients when compared to controls. Curiously, Vit C levels increased after corneal crosslinking, which suggests a compensatory mechanism in the wounded cornea [53].

Vitamin A (Vit A) deficiency was also suspected to contribute to KC development [55]. Recently, it has been shown that retinoic acid supplementation, an active metabolite from Vit A, promotes corneal crosslinking by upregulating transglutaminase-2 (TG2) expression in corneal epithelium and keratocyte [56].

Other vitamins, such as B12 and B9, were proposed to be involved in KC pathogenesis, since they can indirectly affect the activity of collagen synthesis enzymes through homocysteine metabolism. However, serum levels of both vitamins are unaltered [45,57].

### 3.2. Minerals

Nutritional deficiency can also affect the concentration of metal ions, such as copper (Cu), iron (Fe), selenium (Se), and zinc (Zn). These are all cofactors of enzymes involved in collagen synthesis, crosslinking, or antioxidant activity. Therefore, an imbalance in these minerals can be an independent risk factor for KC development [58].

In line with this hypothesis, KC patients display lower serum levels of Cu, Se, and Zn [46,59,60,61]. Moreover, Cu deficiency can be exacerbated in KC by increased tear alkalinity. This would inhibit transfer of Cu ions to the center of the cornea [62], thus generating a localized deficit of Cu. In this vein, peripheral Cu deposition is a common clinical feature in KC patients (i.e., Fleischer ring) [62,63,64]. The mechanism(s) through which deficiency of metal ions may promote KC formation are manifold. Some relevant examples are the Cu-dependent lysyl oxidase (LOX), which promotes collagen crosslinking [65], antioxidant enzymes such as Cu,Zn-Superoxide dismutase (SOD1 and SOD3) [60,61,66,67] and Se-dependent glutathione peroxidase (GPx) [66], and Zn-dependent matrix metalloproteinases (MMPs), which regulate collagen degradation [60].

Dysregulated iron metabolism has also been hypothesized in KC corneas. In fact, Fe has been detected, together with Cu, in the corneal Fleischer ring [62,63]. Due to Fe peripheral deposition, it was hypothesized that central unavailability of Fe contributes to KC development, because it works as a cofactor of lysyl hydroxylase (LH), which is required for the collagen crosslinking process. While no significant differences were found in the serum levels of Fe [59], KC patients show lower levels of iron-binding proteins serotransferrin and lactoferrin in tears [47,68] and the corneal epithelium [69], suggesting that iron homeostasis is locally altered in KC cornea. Interestingly, certain polymorphisms of the transferrin gene have been identified as risk factors for KC [70]. Deranged iron metabolism could contribute to KC development not only by reducing the function of crosslinking enzymes, but also by producing reactive oxygen species (ROS) through the Fenton reaction [71]. This may result in oxidative stress, a well-established cause of KC development.

### 3.3. Hormones

#### 3.3.1. Thyroid Hormones

The contribution of thyroid hormones is still under scrutiny. Some studies have reported a higher thyroid gland dysfunction prevalence among KC patients [72,73], but others failed to prove an association between them [74,75,76]. Nevertheless, increased levels of thyroxine hormone (T4) were found in tears [72,77] and aqueous humor [78]. Moreover, the expression of thyroxine receptor (T4R) was elevated in keratocytes of KC patients compared to controls [72], suggesting an active role of T4 in the pathophysiology of KC.

#### 3.3.2. Sex Hormones

The imbalance of sexual hormone levels has been studied in KC patients. However, data are heterogeneous, and it is still unclear when (age and disease stage) those hormones impact on KC corneas or which molecular pathways are involved [79]. A longitudinal evaluation of middle-aged KC patients failed to show any difference in progression between males and females, or in females with or without hormone treatment [80]. Interestingly, KC typically develops in puberty with stabilization after 40 years of age [9,81]. This is consistent with the profile of significant hormonal changes, supporting a potential role in KC pathophysiology. Lastly, there are reports of KC development and progression during or immediately after pregnancy [82,83].

Some studies have reported an increased expression of androgen [84,85] and estrogen [85] receptor in corneal epithelium retrieved from KC patients, while progesterone receptor showed significantly lower expression [84]. Interestingly, the levels of salivary and plasma estriol and estrone were downregulated in KC patients when compared to controls, while dehydroepiandrosterone sulfate levels were upregulated [53,86,87]. In addition, an alteration of the luteinizing hormone/follicle-stimulating hormone ratio was observed in KC patients, suggesting a role for gonadotropins in KC pathogenesis [88]. Lastly, prolactin-induced protein (PIP), secreted by a number of normal apocrine cells, has been proposed as a marker for KC progression [78,86], as it is altered in several body fluids (e.g., aqueous humor, tear fluid, plasma, saliva) of KC patients.

### 3.4. Metabolites

#### 3.4.1. Redox Metabolism

The redox metabolism involves the broad range of biochemical reactions that maintain the balance between oxidant and antioxidant compounds [89]. When this balance shifts toward the pro-oxidant species, oxidative stress takes place, which may result in massive cell damage [90,91]. The eyes, particularly the cornea, are constantly exposed to environmental stressors, such as UV radiation, pollutants, and injuries. For this reason, enzymatic and nonenzymatic antioxidants are highly expressed in the human cornea [92]. In fact, alterations of the redox balance are involved in several corneal diseases, including KC [93]. The role of redox metabolism alterations in the pathogenesis of KC has been extensively studied [94]. It has been shown that KC corneas display increased production of reactive oxygen and nitrogen species (ROS and RNS, respectively) [95,96]. As a consequence, large amounts of cytotoxic byproducts from both lipid peroxidation (e.g., malondialdehyde) and nitric oxide pathways (e.g., nitrotyrosine, peroxynitrites) were observed [95,97]. In addition, mitochondrial DNA damage has been reported [98].

A possible explanation for the increased oxidative stress is a reduction in the total antioxidant capacity of the KC cornea. In line with this hypothesis, levels of non-enzymatic antioxidants (e.g., glutathione) [54,97,99] and antioxidant enzymes, including SOD and aldehyde dehydrogenase (ALDH) [94,98,100,101], are reduced in KC corneas. Another indicator of oxidative stress is the increase in lactate/pyruvate (L/P) ratio, which was found in both in vitro [102] and in vivo [99] studies.

Interestingly, redox imbalance was not restricted to the ocular surface. In fact, systemic alterations of redox homeostasis were demonstrated in KC patients. Specifically, serum ROS levels were increased, while the activity of antioxidant enzymes (SOD, GPx, catalase) and glutathione levels were reduced in KC patients [66,67,103]. These findings support the hypothesis of systemic metabolic changes that may contribute to KC initiation and progression.

In conclusion, the increased levels of ROS and RNS and the concomitant decrease of the antioxidant defenses leave the cornea more susceptible to oxidative damage and to the degradation of the extracellular matrix, which could result, clinically, in KC corneal thinning [104].

#### 3.4.2. Arachidonic Acid Pathway

Arachidonic acid (AA) is a polyunsaturated fatty acid that is implicated in several biological functions. Due to its four *cis* double bonds, AA helps in maintaining cell membrane fluidity and ability to interact with proteins [105]. Moreover, AA can be metabolized by phospholipase A_2_s (PLA_2_s), cyclooxygenases (COXs), and lipoxygenases into prostaglandins and leukotrienes, important proinflammatory mediators [106]. The AA pathway is involved in different pathophysiological processes in the eye [107]. In particular, an abnormal activity of AA-related metabolic enzymes (e.g., COXs) and of its derived products has been reported in in vitro models of KC [108,109,110]. These findings were confirmed by further studies on KC patients, in which high serum levels of prostaglandin F2α, A2, and E2, as well as 5-hydroxyeicosatetraenoic acid, were reported [111]. These observations are recently leading many authors to consider KC as a *systemic* inflammatory disease [112].

#### 3.4.3. Tricarboxylic Acid Cycle

The tricarboxylic acid cycle, also known as the citric acid cycle or Krebs cycle, is an amphibolic series of chemical reactions occurring in respiring organisms that eventually lead to the production of energy [113]. Defects in this cycle are associated with pathological conditions in different body sites, including the eye [114,115]. Metabolic imbalances involving the citric acid cycle have been reported in KC patients and are confirmed by detection of increased lactate production [53,87,102]. This has deep implications for the cell fate, since abundant lactate synthesis (anaerobic respiration) results in lower extracellular pH, increased oxidative stress, and apoptosis [116]. For these reasons, the lactate/malate ratio has been proposed as an indicator of oxidative stress, which may play a role in KC progression [96,102]. Interestingly, it has been shown that in vitro models of KC receiving collagen crosslinking respond with a shift toward aerobic respiration, with marked increase in ATP synthesis [53].

On the contrary, metabolomics analysis of KC patients’ tears revealed a strong increase of ATP, malate, and malonyl-CoA compared to controls [99]. This difference could be due to the contribution of other ocular surface structures (e.g., conjunctiva, lacrimal glands) to the tear composition.

#### 3.4.4. Glycolysis and Gluconeogenesis

Glycolysis and gluconeogenesis are two metabolic pathways involved in glucose catabolism and anabolism, respectively, which play a key role in cell survival [117,118]. Studies performed on in vitro models of KC demonstrated that glucose metabolism is upregulated [119,120,121]. In particular, anaerobic glycolysis appears to be the most affected, since concentrations of lactate are higher than pyruvate [102]. Interestingly, it was shown that sex hormones are able to alter glucose metabolism in human keratoconus cells (HKCs), a finding that is corroborated by the observation that KC typically develops in puberal age [122,123]. Metabolomic analysis of tears from KC patients showed a significant increase in metabolic intermediates involved in anaerobic glycolysis, confirming the in vitro results [99].

Findings on glucose metabolism alterations led to the investigation of a possible role of diabetes mellitus (DM) in KC pathogenesis. However, results obtained from different studies were controversial, and clinical data suggest that DM is a protective rather than a risk factor for KC onset and progression [121,124].

#### 3.4.5. Urea Cycle Metabolism

The urea cycle, also known as the ornithine cycle, is a human metabolic pathway, which serves for ammonia detoxification [125]. Stimulation of HKCs with dehydroepiandrosterone (DHEA), a sex hormone upregulated in the serum of KC patients, directly upregulated urea cycling [111]. This stimulation led to an altered availability of precursors necessary for the biosynthesis of proline and hydroxyproline, two major components of collagen [123], thereby altering collagen metabolism. Similarly, the metabolomic signature from tears of KC patients showed low levels of ornithine and an upregulation of aspartate, supporting an imbalance in the urea cycle [99].

#### 3.4.6. Fatty Acid Metabolism

Lipids and fatty acids are two of the major components of the human cornea. They are involved in different cellular pathways, modulating pro- and anti-inflammatory reactions, promoting corneal tissue proliferation and repair, and contributing to neovascularization [126]. The most representative fatty acids in the human cornea are oleic, stearic, and palmitic acids [127]. In a recent study, gas chromatography and mass spectrometry were used to define a metabolomic signature to discriminate between healthy and KC corneas [128]. A significantly reduced amount of both saturated fatty acids, such as stearic, palmitic, myristic, and pentadecanoic acid, and unsaturated fatty acids (e.g., linoleic and *trans*-13-octadecenoic acid) was reported. Lower levels of fatty acids in KC patients may be explained by the reduced amount of malonyl CoA observed in previous studies [99,129]. In fact, malonyl CoA is an essential precursor for fatty acid biosynthesis [130].

## 4. Conclusions

The existing literature supports a role for many nutrients, hormones, and metabolites in KC pathogenesis. The proposed mechanisms of action are manifold, but generally converge toward the promotion of collagen degradation and inhibition of its synthesis/crosslinking. It remains unclear, however, if these alterations are the primary cause of KC or, rather, if they are consequent to the deeply altered KC metabolome.

It should be noted, however, that existing data support the existence of systemic, rather than eye-isolated nutritional and/or metabolic imbalance. Interestingly, some of these alterations have been associated with higher prevalence or worse prognosis. In this context, it is tempting to speculate that these alterations may translate into predictive/monitoring biochemical markers measured in the tear fluid/peripheral blood. Perhaps more importantly, the time has come to consider KC as a systemic disease, where nutritional/metabolic imbalances may play a role.

## Data Availability

Not applicable.
